# A phenotypic screening platform to identify small molecule modulators of *Chlamydomonas reinhardtii *growth, motility and photosynthesis

**DOI:** 10.1186/gb-2012-13-11-r105

**Published:** 2012-11-18

**Authors:** Simon E Alfred, Anuradha Surendra, Chris Le, Ken Lin, Alexander Mok, Iain M Wallace, Michael Proctor, Malene L Urbanus, Guri Giaever, Corey Nislow

**Affiliations:** 1 Donnelly Centre for Cellular and Biomolecular Research, University of Toronto, 160 College Street, Toronto, Ontario M5S 3E1, Canada; 2 Banting and Best Department of Medical Research, University of Toronto, 112 College Street, Toronto, Ontario M5G 1L6, Canada; 3 Department of Molecular Genetics, University of Toronto, 1 King's College Circle, Toronto, Ontario, M5A 1A8, Canada; 4 Department of Pharmaceutical Sciences, University of Toronto, 144 College Street, Toronto, Ontario M5S 3M2, Canada; 5 Stanford Genome Technology Center, Palo Alto, CA 94304, USA; 6 Novartis, 250 Massachusetts Ave., Cambridge 02139, USA

## Abstract

Chemical biology, the interfacial discipline of using small molecules as probes to investigate biology, is a powerful approach of developing specific, rapidly acting tools that can be applied across organisms. The single-celled alga *Chlamydomonas reinhardtii *is an excellent model system because of its photosynthetic ability, cilia-related motility and simple genetics. We report the results of an automated fitness screen of 5,445 small molecules and subsequent assays on motility/phototaxis and photosynthesis. Cheminformatic analysis revealed active core structures and was used to construct a naïve Bayes model that successfully predicts algal bioactive compounds.

## Background

Chemical biology uses small molecules to study and manipulate biological systems (reviewed in [1]). By altering an organisms' normal state, and thereby affecting growth or development, we can learn about the contributions of the perturbed processes to the organisms' fitness, physiology and homeostasis. The approach is analogous to genetic manipulation to produce an observable phenotype (reviewed in [2]). Small molecules, in addition to complementing genetic perturbations, have several advantages: they can be applied at varied concentrations, during different stages of development and on specific tissues, to different organisms, and their (frequently) rapid reversibility can be used to modulate dynamic processes.

The single-celled green alga *Chlamydomonas reinhardtii*, often referred to as the 'green yeast' [3], is a powerful model organism that can be easily manipulated, with straightforward genetics and a wide range of informative phenotypes. Furthermore, its biology is relevant to both plants and animals and to human disease, for example, ciliopathies [4,5]. Key discoveries have been made from study of *Chlamydomonas*' chloroplast [6,7] as well as components of its light perception [8] and light response complexes [9]. Evolutionary studies have focused on evolution of fitness under selective pressures (elevated [CO_2_], sexual/asexual populations) and the evolution of multicellularity [10-13]. *Chlamydomonas *has provided a wealth of data on flagellar formation and function and intraflagellar transport was first observed in *Chlamydomonas *[14]. As one of the few model organisms with motile flagella, combined with renewed interest in primary cilia and flagellar/ciliary disorders [15], *Chlamydomonas *is a clinically important test system for perturbation.

Chemical perturbation studies on green algae have focused primarily on inhibitors of photosynthesis, particularly for agricultural and research applications [16,17]. *Chlamydomonas*' chemical sensitivity has also been exploited to assess environmental toxins such as cadmium and the herbicide fluoxypyr [18,19], providing information on small molecule bioaccumulation and uptake [20,21]. In addition, chemical genetic screens have tested small molecules for their ability to alter motility [22,23], phototaxis [24], and flagellar formation and regeneration [25,26]. However, a large-scale, comprehensive screen for small molecule inhibitors of fitness has, to our knowledge, not yet been reported. Our experience with yeast chemical perturbation has shown that data derived from simple fitness screens are quite valuable, with growth being the ultimate 'integrative phenotype'. Information at this stage can be invaluable for facilitating genetic and genomic approaches to determine possible mechanisms of action of small molecule growth inhibitors [27,28].

Here we developed a small molecule screen at very high-throughput to identify fitness inhibitors of *C. reinhardtii*, based on two screens, one for long-term fitness in the presence of compound, and another for phenotypic effects on photosynthesis and motility/phototaxis using short-term exposure. We demonstrate the effectiveness of *Chlamydomonas *as a chemical biology subject, and define and model physiochemical parameters that characterize small molecule activity on *Chlamydomonas*. As part of this study we generated a chemical biology *Chlamydomonas *resource searchable by identifier or structure and that provides detailed growth and phenotypic metrics of small molecules on *Chlamydomonas *[29].

## Results

### *Chlamydomonas *as a chemical biology model

**Figure 1:**
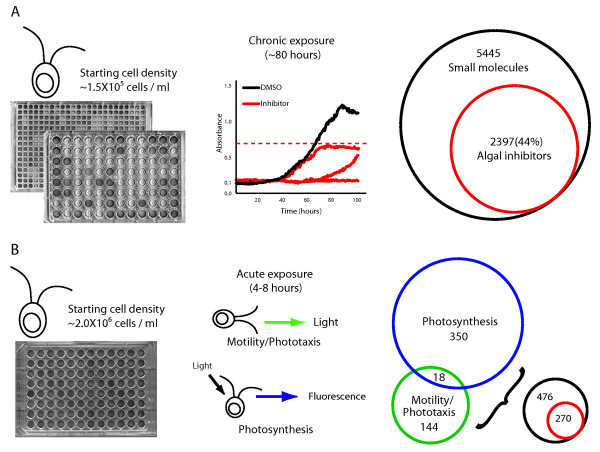
***Chlamydomonas reinhardtii *small molecule screens**. **(a) **Chronic fitness screen: 96- and 384-well microtiter plates were inoculated with low density cells (1.5 × 10^5 ^cells/ml) and small molecules were added to a final concentration of 25 µM (0.5 µg/ml FDA library). Growth was monitored by optical density in our Freedom Evo robotics platform every 30 minutes, resulting in detailed growth curves. A total of 5,445 small molecules were screened with 44% inhibiting *Chlamydomonas *growth by 35% or more compared to in-plate controls. **(b) **Acute exposure screen: 96-well microtitre plates were prepared similarly to the chronic screen except with a higher cell density (2 × 10^6 ^cells/ml). Plates were assayed for alterations in motility utilizing a phototaxis assay or photosynthesis by assaying fluorescent induction. Of the 476 unique acute inhibitors, 45% were also active in the chronic exposure screen.

To screen for small molecules that inhibit the growth of *Chlamydomonas *we performed an 80-hour fitness assay (Figure 1a). Our assay takes advantage of tools developed for yeast growth that monitor optical density (OD) of individual microtiter plate wells several times each hour, resulting in high resolution growth curves [27,30]. This high-throughput method combines a liquid handling robot, plate reader and integration software [30]. Growth was performed at constant temperature (22°C), agitation (150 rpm) and illuminance (40 µmol photons/m^2^s) and was validated in both 96- and 384-well plates.

To determine conditions for small molecule screening on *Chlamydomonas *we performed growth assays with solid and liquid TAP media with varying concentrations of solvent (DMSO) or small molecules. DMSO is a preferred diluent because the majority of available chemicals are soluble in DMSO at high concentrations and many chemical libraries are pre-plated in DMSO. We tested CC-125, a common laboratory strain, in DMSO doses ranging from 0.25 to 2.5%, in liquid and solid media to determine a dose for screening (Figure S1 in Additional file 1). In liquid TAP media DMSO concentrations above 1.0% (v/v) had adverse effects, with cells collecting at the bottom of wells, and 2.5% severely reduced growth. On solid media DMSO was deleterious to growth at concentrations greater than 2.0%, at which point cells became swollen and chlorotic. Based on these tests, we selected a concentration of 0.5% DMSO for liquid TAP and 1.0% for solid TAP media [16,31,32].

We next assayed the fitness of *Chlamydomonas *by light scattering (OD_600_) in 96- and 384-well plates to determine the cell density that provided the most reproducible high-resolution growth curves with the greatest dynamic range. Suitable volumes for each plate type were determined empirically, 200 µl for 96-well plates and 70 µl for 384-well plates. In addition, we miniaturized *Chlamydomonas *fitness assays further by screening 9 µl in 1,536-well plates (Figure S2 in Additional file 1).

In a pilot study, we screened 168 novel small molecules (plates 723N5803 and 723N5890 from the Chembridge Novacore collection [33]) at doses ranging from 25 to 200 µM to determine a suitable screening dose. Virtually all cells died at concentrations above 100 µM regardless of structure (data not shown). Compared to *Saccharomyces cerevisiae*, *Chlamydomonas *is significantly more sensitive to small molecules. We chose an initial screening dose of 25 µM because it yielded the best balance of inhibition without being toxic. This dose is similar to that used in small-scale screens on *Chlamydomonas*, plants and zebrafish [16,24,32,34,35].

Although we anticipated that the hydroxyproline-rich cell wall of *Chlamydomonas *could present a physical barrier for small molecule uptake [36]; based on the efficacy of our tests and those in the literature, *Chlamydomonas *is sufficiently permeable to diverse chemical structures. To directly evaluate small molecule permeability on *Chlamydomonas*, we quantified the intracellular accumulation of exogenously added chemicals using high-pressure liquid chromatography (HPLC). Briefly, *Chlamydomonas *cells were treated with select small molecules for 2 to 4 hours, washed and lysed, and lysate was separated via high-pressure liquid chromatography (HPLC) [37]. We found that drugs accumulate in a dose-dependent manner, based on the appearance of a peak in treated samples that elutes at a similar time and with the same spectral qualities as the drug in buffer (Figure S3 in Additional file 1). Our results indicate that small molecule uptake and accumulation in *Chlamydomonas *is more efficient than in *Caenorhabditis elegans *and *S. cerevisiae *[37,38].

We tested the growth of wild-type strains, non-motile mutants, as well as mutants that display altered phototaxis or photosynthesis, in our automated fitness assay to compare growth curves among the different strains (Table S1 in Additional file 2). All strains tested show comparable growth dynamics compared to wild-type (CC-125); however, motility mutants, *bld2 *(CC-478) and *pf14 *(CC-1032), occasionally show jagged growth curves, suggesting that their lack of motility can cause cells to aggregate and/or pool at the bottom of wells, which could result in erratic OD readings from one time point to the next. To minimize these effects plates are shaken at 150 rpm during growth.

### *Chlamydomonas *small molecule screens

We screened *C*. *reinhardtii *(CC-125) for sensitivity to compounds against several commercially available chemical collections, including the 'FDA library', a collection of 640 approved and off market drugs (Enzo Life Sciences, Farmingdale, NY, USA); 'Tim Tec', a 280 member natural product library consisting of herbicides and natural extracts (TimTec LLC, Newark, DE, USA); and the 'yactives library', a novel small molecule collection that we collated based on yeast growth inhibition from ChemBridge (NOVACore and DIVERSet) and ChemDiv (Divers) collections [38] (Table S2 in Additional file 2). In total we screened 5,445 unique small molecules in triplicate (with the exception of the TimTec library because of limited amounts and two NOVACore plates from the pilot screen). We calculated the standard deviation to assess variation between replicates and developed a compound activity score based on the average of the triplicate data. Actives were scored based on the area under each growth curve versus an in-plate DMSO control. Compounds that resulted in a growth ratio of 0.65 or less (that is, 35% inhibition), defined *Chlamydomonas *fitness inhibitors. Using this metric, 44% (2,397) of small molecules from our screen of 5,445 distinct small molecules altered algal growth (Figure 1a). This enrichment is quite high, and likely reflects that the majority of these small molecules have been pre-selected for bioactivity. Indeed, we have shown that compared to random compounds, the yactive library is enriched 3- to 12-fold when screened against *Escherichia coli*, whole worms and cultured mammalian cells [38].

To complement the fitness screen, we performed an acute exposure screen to measure light- and motility-related phenotypes. By treating *Chlamydomonas *for hours (as opposed to days) we can observe immediate effects of chemical activity on cells within a single cell cycle. We assayed motility/phototaxis and photosynthesis because they are well-studied phenotypes and are amenable to detailed downstream follow-up analysis (Figure 1b). These screens were performed similar to fitness screens, but with a higher starting cell density (2 × 10^6 ^cells/ml versus 1.5 × 10^5 ^cells/ml) and for a shorter time (4 to 8 hours versus approximately 80 hours). Phototaxis was evaluated by *Chlamydomonas*' response to a strong directional light; 3 minutes following light exposure an image was captured and the response scored. We tested photosynthetic efficiency by assaying fluorescence induction at 685 nm, the fluorescent maxima for photosystem II (PSII). Specifically, plates were transferred from low light into a plate reader, shaken at 100 rpm in the dark for 20 seconds and then excited with 470 nm light, an optimal wavelength for photosynthetic function. In this procedure a higher fluorescence signal corresponds to reduced photosynthetic capacity, because the emitted light is released as fluorescence rather than being used for photosynthesis [39].

From the 5,445 chemicals screened in the chronic assay, 4,841 (88.9%) were screened in the acute assay. In this latter set of compounds we identified 144 motility/phototaxis modulators and 350 photosynthetic inhibitors, of which 18 were found in both assays (Figure 1b). Of these 476 small molecules active in these acute screens, 270 were also growth inhibitors, suggesting that their growth inhibition may be a result of modulating components involved in motility/phototaxis or photosynthesis, or alternatively, by secondary effects on essential cellular processes. Motility/phototaxis modulators were separated into two classes, 106 inhibitors of motility (no response to directional light) and 38 modulators of phototaxis sign (altered response to directional light). Vinpocetine, a phosphodiesterase and Na^+ ^channel inhibitor [40,41], is a phototaxis modulator resulting in positive phototaxis in our assay (untreated cells display negative phototaxis; Figure S4 in Additional file 1), which likely modulates phototaxis by altering concentrations of Na^+ ^and Ca^2+ ^ions.

Following the acute and chronic exposure screens we pinned the cells in each well onto TAP agar without compound to determine which chemicals under each treatment were cytocidal or cytostatic. We performed the cytocidal/cytostatic assay for cells treated in the fitness screen (approximately 80 hours) and found that 11.5% are cytocidal and 33.2% are cytostatic. The difference in cytocidal/cytostatic percentages between the chronic and acute assays suggests that exposure time is critical, and that most small molecules under short-term exposure are reversible. However, we found that only 0.9% (44/4,841) of chemicals are cytocidal and 0.3% (17/4,841) are cytostatic under the short exposure of the acute screen (4 to 8 hours). The results from the acute screen indicate that fitness inhibitors also show effects in our acute exposure experiments and demonstrate that the fitness assay identifies inhibitors of specific, growth-dependent processes.

**Figure 2:**
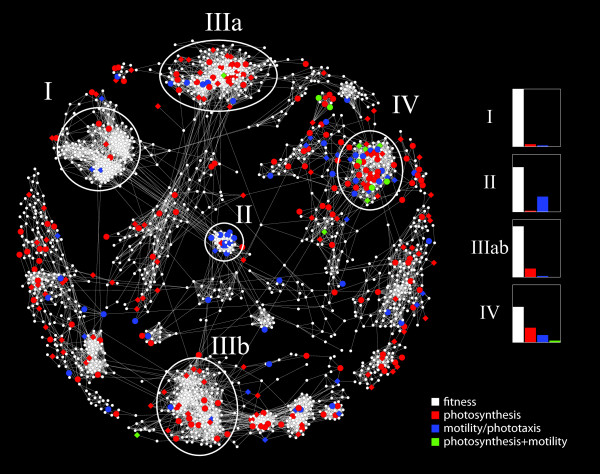
**Network view of active molecules**. All active small molecules were clustered according to their ECFP_4 chemical fingerprints. Each node represents a unique small molecule: 1,802 small molecules cluster together and are shown here. Edges represent structural relatedness at a cutoff of ECFP_4 greater than 0.5. Core structural clusters are outlined and designated groups I to IV. Acute screen data were mapped onto the active network with white fitness inhibitors, red photosynthetic inhibitors, blue motility/phototaxis modulators, and green photosynthetic and motility/phototaxis; circles indicate fitness inhibition, diamonds indicate no fitness inhibition. Bar graphs: phenotypes for each chemical group are displayed as a percentage.

To gain insight into the physiochemical properties that confer activity on *Chlamydomonas*, we clustered all active small molecules by their structural similarity and mapped them on each phenotype (fitness, motility/phototaxis, and photosynthesis) to define substructures enriched for specific activities. We calculated ECFP_4 similarity [42] for each small molecule versus the entire 5,445 screened collection and using Cytoscape [43] and a cutoff of 0.5 (on a scale of 0 to 1.0, with 1.0 representing identical compounds), we clustered and visualized the data using a network topology (Figure 2). We then mapped our phenotypic data using Cytoscape onto our small molecule network to identify clusters that group according to structure and phenotype.

Several major structural classes of compounds are highlighted to illustrate that related structures cluster with specific phenotypes (Figure 2and Table 1). We highlighted five chemical clusters (groups I to IV) based on their phenotypic activity profile. Fitness inhibitors are by far the largest class of small molecule identified in our screens. Group I inhibitors, characterized by a shared benzyl-methyl-phenylethyl-piperidinyl-methylamine, predominately inhibit growth. Group II compounds affect motility/phototaxis, which appear to inhibit motility as a result of their high toxicity, as cells in the phototaxis assay show no response to directional light and most treated cells are lysed. We found that photosynthetic inhibitors are found evenly across our network, indicating that diverse chemical structures can alter photosynthesis. Not surprisingly, there is significant overlap between photosynthesis and growth inhibitors - for example, herbicides affect growth based on their effects on photosynthetic efficiency. Group III is the combination of two unrelated clusters, phenyl-piperidine-oxazoles and benzoyl-piperidine, enriched for photosynthetic inhibitors. To assess the mode of action of these photosynthetic inhibitors, we screened a subset of the 350 photosynthetic inhibitors on a well-characterized DCMU resistant strain, which has the V>I mutation in the D1 protein at residue 219 [44]. Based on the resistance of the mutant to uncharacterized photosynthesis inhibitors, we can gain insight into their mode of action and prioritize novel photosynthetic inhibitors for follow-up. Among 48 active photosynthetic inhibitors we found 23 chemicals that affect wild-type (CC-125) but not a DCMU-resistant line (CC-1403), suggesting the D1 mutation confers resistance to many compounds within our set of uncharacterized inhibitors. Group IV small molecules possess activity in all phenotypes tested, indicating the biphenyl-pyrazole substructure is particularly active. Interestingly, most chemicals that reverse *Chlamydomonas *response to light are from group IV.

**Table 1:** Groups I to IV core chemical structures and reported activities

Core structure group	Phenotype	PubChem bioassays (AID)	Named drugs and uses
I	Fitness	PubChem AID 317390, 317391, 317396, 317399, 317400, 5512, 52703; displaces binding of sigma receptor, binds sigma receptor, binds 5HT-2A receptor, inhibits chemokine receptor 3 (CCR3)	
II	Motility/Phototaxis	PubChem AID31792; acetocholinesterase inhibitor	Cyclizine, antihistamine; benethamine,
IIIa	Photosynthesis	PubChem AID1865, 2314, 2315, 2546, 504333, 493056; epigenetic regulator, shiga toxin inhibitor, inhibits retinoic acid related orphan receptor gamma, inhibits BAZ2B, increases thyrotropin releasing hormone receptor	Darglitazone, muraglitazar, peroxisome proliferation activated receptor gamma, antiglycemic; oxaprozin, NSAID
IIIb	Photosynthesis	PubChem AID894, 1529, 145655, 5512; binds 5HT-2A receptor, inhibits MEK5 kinase, inhibits 15-hydroxyprostaglandin dehydrogenase	Tolperisone, muscle relaxant
IV	Active in all phenotypes	PubChem AID265123, 265124, 265125, 265126, 265127; binds dopamine receptors D2, D3, D4, D1A	Fezolamine, antidepressant; lonazole, NSAID, COX2 inhibitor

### A structural model for *Chlamydomonas *bioactive compounds

**Figure 3:**
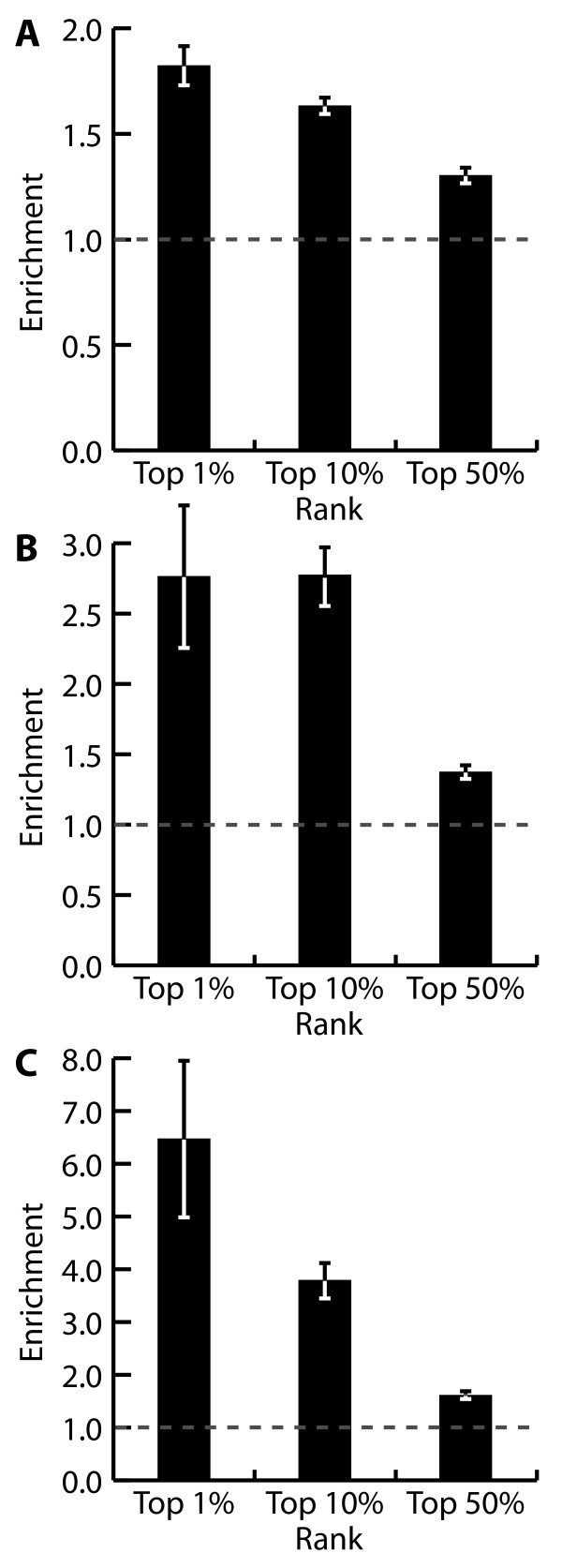
**Naïve Bayes modeling for active small molecule prediction**. **(a) **Fitness model. **(b) **Photosynthesis model. **(c) **Motility/phototaxis model. (a-c) The model was trained on four-fifths of the active set and tested against the remaining fifth. Assessment is repeated five times and compared to random selection of active small molecules to calculate the model enrichment factor. Error bars represent standard error of five replicates.

Using chemical fingerprinting analysis of the active versus inactive small molecules, we constructed a model to predict active algal inhibitors. Chemical structures of active and inactive small molecules were analyzed using the ECFP_4 chemical fingerprinting [42], and were used to train a naïve Bayes model to determine chemical groups over-represented in the active class. To test the performance of the model we trained it using four-fifths of the dataset and then predicted actives in the remaining fifth of the data. This was repeated five times to ensure that each fifth of the data was used as the comparison set. We then generated one model for each phenotype to predict compounds that were active in fitness, motility/phototaxis, and photosynthetic structures. Using these models we ranked small molecules by predicted algal activity and compared the number of tested algal active compounds identified versus a randomly ordered set. At a predictive rate of 10% (selecting the top 10% of ranked compounds versus a random selection), the fitness, motility/phototaxis, and photosynthetic models showed enrichment for actives of 1.6×, 3.8×, and 2.8×, respectively (Figure 3a-c). These enrichments could easily save significant costs in labor and time (for example, screening 10% of a 10K library with a hit rate of 44% results in 440 hits randomly versus 704 hits from a prioritized list with an enrichment of 1.6×). We next used our model to predict algal actives on a 50,000-member small molecule library (NOVACore, ChemBridge, San Diego, CA, USA) and empirically tested 253 of the predicted compounds. Our model accurately predicted active small molecules in the unscreened library, enriching for algal fitness inhibitors 2.8× over a non-prioritized set of the same library, demonstrating the utility of such predictive models for prioritizing molecules for screening.

### Human antipsychotics alter *Chlamydomonas *motility

Our screen included two small molecule collections of known inhibitors, the 'FDA and TimTec' libraries, of which approximately 7% (61) are algal actives. These compounds were enriched for chloroplast and mitochondria targets, and antifungals including ergosterol inhibitors, and human antipsychotics. The antipsychotics were enriched for those involved in dopamine and serotonin signaling. Intriguingly, we found that a majority of our chemical groups (Figure 2) have activity in diverse dopamine and serotonin assays (Table 1).

**Figure 4:**
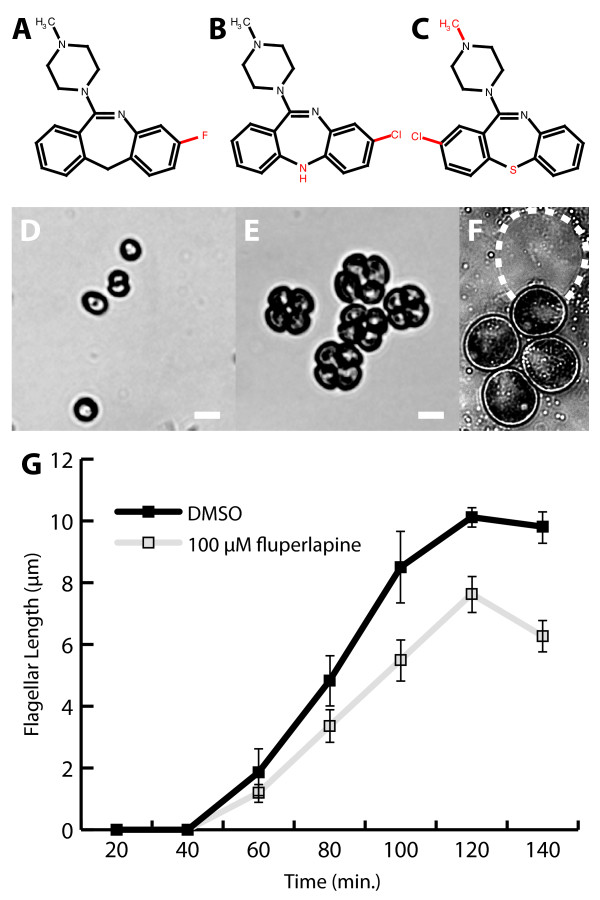
**Effects of fluperlapine on *Chlamydomonas***. **(a-c) **Dibenzazapine analogs: (a) fluperlapine; (b) clozapine; (c) clothiapine. Common core structure is shown in black, variable regions in red. **(d) ***Chlamydomonas *cells grown in TAP with 0.5% DMSO. **(e) **Cells grown in TAP with 25 µM fluperlapine. **(f) **Fluperlapine-induced cell cluster produces four distinct flagella-less cells upon applied pressure. Parental wall is outlined in a dashed white line. **(g) **Flagellar regeneration of *Chlamydomonas *in solvent (closed squares) or 100 µM fluperlapine (open squares). Length of at least 20 flagella per time point, averaged from two independent replicates is plotted. Error bars represent standard error. Scale bar 10 µm.

To better understand the activity of antipsychotics on *Chlamydomonas *we focused on the atypical antipsychotic dibenzazepines because of their activity in our screens, therapeutic relevance and availability of structural analogs. In our dataset this cluster is composed of structural analogs of clozapine, including clothiapine (Figure 4a-c), an active algal growth inhibitor in our assay, and fluperlapine, which we found caused cell pooling in the acute exposure screen but does not affect fitness at screening doses. Interestingly, clozapine, which differs from fluperlapine by one atom, does not show growth inhibition at the same concentration. When a broad range of concentrations was tested, clozapine and other analogs, loxapine, quetiapine, and clothiapine, produced a similar aggregation phenotype as fluperlapine and were all inhibitory at high concentrations, suggestive of different potencies but similar modes of action.

To further characterize the dibenzazepine effect, cells following fluperlapine treatment were observed microscopically (Figure 4d, e). Two predominant classes of cells were observed; large transparent cells (36/80) and small clusters of typically four cells (31/80). The large cells were chlorotic with vacuolated chloroplasts, indicating these cells were dead or dying. The cells within the four-cell clusters appeared normal, but remained encased in a parental cell wall. Mechanical stress released four aflagellate cells, indicative of a hatching defect (Figure 4f). Because hatching requires the cell wall degrading enzyme sporangin, which is expressed only in the flagella [45], aflagellate cells (that is, bld-2) have difficulty hatching [45]. Given the phenotypic similarities between fluperlapine treatment and flagellar mutations, we speculate that fluperlapine and its analogs could modulate flagellar growth/development to prevent hatching.

To determine if fluperlapine affects flagellar growth, we performed a flagellar regeneration assay in the presence of fluperlapine. Several inhibitors are known to shorten flagella, including IBMX, colchicine and cytochalasin D [46,47]. We deflagellated cells using the pH shock method [48] and assayed flagellar length every 20 to 30 minutes (Figure 4g) in the presence of 100 µM fluperlapine. This dose resulted in an observable effect, yet did not affect viability after washout. Over the course of approximately 2 hours, flagella regenerated to near pre-deflagellation lengths (8.97 ± 0.5 µm; pre-deflagellation 10.9 ± 0.7 µm) in solvent-treated cells, while fluperlapine-treated cells regenerated to just over half length (7.0 ± 0.5 µm). We also treated cells with several doses of fluperlapine and clozapine for 12 hours. Fluperlapine-treated cells (50 µM) had flagella (7.2 ± 0.4 µm), similar to that observed in the flagellar regeneration assay; however, cells treated with clozapine (50 and 100 µM) and high concentrations of fluperlapine (100 µM) were aflagellate and inviable.

## Discussion

In this study we demonstrate that high-throughput screening of the model alga *C. reinhardtii *is an effective means to identify novel small molecule probes. Our work builds on previous studies focused on small molecule herbicides and well-characterized inhibitors and combines it with high-throughput methods developed for bacteria and yeast. For example, the PSII inhibitors DCMU and atrazine, and other modulators of photosynthesis, have contributed to our understanding of photosynthesis and signaling [44,49]. Antibiotics that target the chloroplast were instrumental in demonstrating uniparental chloroplast inheritance [6]. Research on motility and the cell cycle sparked interest in various classes of modulating compounds, including microtubule inhibitors [50,51], many of which can discriminate between animal and plant tubulins and are therefore useful in agriculture [52]. Inhibitor studies have also shed light on the biology of flagellar regeneration [46] and microtubule organization [31]. Chemical interrogation of phototaxis using chemical screens has identified modulators of motility and light perception [24,53,54]. In addition, studies on a diverse group of chemical classes, including anesthetics [26,46,55,56], phosphodiesterase inhibitors [23,57], DNA damaging drugs [58], antipsychotics [22], antifungals and translation inhibitors [55,56,59,60], have established the sensitivity of *Chlamydomonas *to diverse chemical classes and structures. Recent efforts have exploited *Chlamydomonas*' small molecule sensitivity to develop it as a biomonitor of pesticide, herbicide and heavy metal accumulation in water systems [19,61].

Our screens are quantitative (providing high-resolution growth curves for each small molecule) and large enough to identify active compounds that can be classified into phenotypic categories. Profiling screens incorporating fitness readouts have shown remarkable predictive ability in binning known inhibitors with small molecule libraries [28,38,62-64].

Using the results from our screens, we generated a model that effectively predicts the *Chlamydomonas *bioactivity of compounds and allows one to rank and prioritize libraries for screening. By training a Naïve Bayes model with chemical fingerprinting data from algal actives we were able to predict fitness inhibitors in an unscreened small molecule library, resulting in enrichment of actives identified by 2.8×. Our model can be improved in an iterative manner, as more data become available for training, increasing its predictive accuracy.

All screens are accessible for individual query or bulk download from our website [29]. This website contains fitness and phenotypic metrics for each small molecule tested, as well as links to several chemical repositories, including PubChem [65], ChemBank [66] and PharmGKB [67]. Also included are several unscreened chemical libraries with predicted activity scores on *Chlamydomonas *as a resource for further screening.

Finally, we identified a class of neuroleptics, clozapine analogs that appear to modulate *Chlamydomonas *flagellar growth/function. In animals, these drugs target serotonin and dopamine receptors; however, such targets are absent in green algae and therefore the pathways affected in *Chlamydomonas *may represent functions that are evolutionarily conserved and ancient. For example, Avasthi *et al*. [22] recently reported results from a *Chlamydomonas *motility screen using the chemical library LOPAC (Library of Pharmacologically Active Compounds), in which they found an enrichment in antipsychotics with reported targeting to G-protein coupled receptors and observed modulation of mammalian ciliary length. Our findings on fluperlapine-treated cells suggest modulation of flagellar growth based on the absence of flagella in chronically grown cells, aberrant hatching and reduced flagellar regeneration.

## Conclusions

The maturation of *Chlamydomonas *as a model 'plant' makes its description as the green yeast more appropriate now than ever before. *Chlamydomonas *genomics, combined with the features that have made it an excellent genetic system, promise applications beyond the laboratory - for example, in biofuel development. A recent observation shows that changes in culture conditions can coax *Chlamydomonas *cells to produce an abundance of triacylglycerol, to the point where it comprises much of the cell volume (reviewed in [68]). The platform we describe here is well suited to (i) systematize molecular breeding experiments and (ii) explore chemical and environmental space to uncover perturbations that produce a desired phenotype. Combining such high-throughput screening capacity with genetic perturbations across the *Chlamydomonas *genome will permit new research insights for understanding 'non-yeast' biology - for example, ciliopathies, photosynthesis, and biofuel precursor production, to name a few. Indeed, recent advances in next-generation sequencing for assessing genome-wide mutant collections (reviewed in [69]) make the development of a systematic *Chlamydomonas *mutant collection (for example, using barcoded transposons or complete deletions [70,71]) not only possible, but essential.

## Materials and methods

### Strains and growth conditions

Screens were performed using the *Chamydomonas *reference strain CC-125 (nit1^-^, nit2^-^; a gift from the Dutcher Lab) grown in TAP media [72] under constant illumination (LED strip light, Lumicrest, Toronto, ON, Canada). Cells for screening were inoculated from pre-cultures in which 4 ml of TAP was inoculated from a single colony, cells were cultured to OD_600_~0.1 for chronic or OD_600_~0.4 for acute screens.

Growth assays were performed in clear, flat-bottom 96- and 384-well microtiter plates (VWR International, Mississauga, ON, USA) sealed with adhesive plate seals (catalogue number AB-0580) using a custom developed platform incorporating microtitre plate reader Safire2 and the Freedom EVO (Tecan-US, Durham, NC, USA). Chronic screens were carried out at an initial cell density of 1.5 × 10^5 ^cells/ml (OD_600_~0.1). A final screen concentration of 25 µM (10 µg/ml FDA library) with a DMSO solvent concentration at 0.5% in a total volume of 190 to 200 µl/well was chosen based on initial testing. We found that concentrations of DMSO greater that 0.5%, in liquid assays, resulted in aberrant growth (aggregating cells; Figure S1 in Additional file 1). Plates were shaken in constant light to saturation at 150 rpm, for 3 to 4 days, with at least four DMSO controls per plate. Algal growth was assayed using a Tecan Safire2 plate reader measuring OD every 30 minutes at OD_600 _nm. OD readings are output to provide detailed growth curves (OD over time) that can distinguish percent variations in growth. We were also able to assess fitness in 1,536-well plates as above. Plates were prepared by inoculating 50 µl of cells with drug or solvent to the appropriate concentration, aliquoting 9 µl to each well, and spinning down at 500 rpm for 10 s to remove bubbles.

Acute screens were prepared as above except they were performed only in 96-well microtiter plates at an initial cell density of 2.0 × 10^6 ^cells/ml (OD_600_~0.4) and incubated between 4 and 8 hours. Phototaxis response was assayed by placing 96-well plates in a strong directional light for 3 minutes. Images for each plate were captured and the assay was repeated at least 15 minutes later on the other side of the plate to improve resolution of wells far from the light. Images were scored for inhibitors of motility, no movement, or movement towards light, the opposite of untreated cells. Photosynthetic efficiency was assayed by fluorescent induction in the Safire2 plate reader. Plates were kept in low light prior to the assay. We assessed photosynthetic efficiency by exciting with 470 nm actinic light and measuring the emission at 680 nm, in which a higher reading reports inhibited photosynthesis. To determine cytotoxicity (cytocidal/cytostatic) we pinned treated cells onto 2% agar in 96-well format and allowed them to grow for 10 days at 24°C before they were analyzed.

### Chemicals and libraries

The chemical libraries screened were FDA BML-640 (Enzo Life Sciences, Farmingdale, NY, USA), Yactives, a prescreened yeast actives set derived from ChemBridge (NOVACore and DIVERSet, San Diego, CA, USA) and ChemDiv (Divers, San Diego, CA, USA), and two stock plates from ChemBridge (NOVACore, San Diego, CA, USA). TimTec NPL-280 (TimTec LLC, Newark, DE, USA) was supplied at 5.0 mM in DMSO and was a gift from D Desveaux (University of Toronto). Lugol's stain was obtained from Sigma (62650-100ML-F, St Louis, MO, USA) and diluted to 10 µM in water. Formaldehyde 10% (04018, Polysciences, Warrington, PA, USA) was diluted to 1% in water. Fluperlapine was ordered from Enzo Life Sciences (BML-NS109, Farmingdale, NY, USA) and suspended in DMSO to a stock concentration of 100 mM.

### Screening chemical libraries on *C*. *reinhardtii *

A compound was considered active on *C*. *reinhardtii *if the area under the growth curve after reaching saturation was less than 65% of the DMSO control (ratio [compound/control] <0.65). Automatic flagging of actives was confirmed by visual inspection of the data. Compounds were added to the culture using a 2 µl or 600 nl pin tool (V&P Scientific, San Diego, CA, USA) for 96- or 384-well microplates, respectively, to dilute the compounds 200 times to a final DMSO concentration of 0.5%.

### Cheminformatic analysis

For all chemicals ECFP_4 similarity was calculated using the cheminformatic package in Pipeline Pilot version 6.1 (Scitegic Inc. Accelyrs, San Diego, CA, USA). Chemicals with a similarity of greater than 0.5 (1.0 being identical) were visualized using Cytoscape version 2.8.2; results from phenotypic screens were added as attributes and were used to alter node shape and color. Marvin version 5.4.1 (ChemAxon, Budapest, Hungary) was used for drawing and displaying chemical structures. Naïve Bayes model building was performed as described [38].

### Flagellar regeneration

Cells were grown for two days in a 16 h/8 h light/dark cycle to an OD_600_~0.4. Cells were deflagellated using the pH shock method [48] by adding 350 µl of 0.5 N acetic acid, inverted for 40 seconds, and neutralized with 125 µl Na2CO3, to 6 ml of culture. Deflagellation was confirmed by observation of cells at 40× using a DMIL inverted light microscope (Leica). Deflagellated cells were aliquoted into 1.5 ml tubes and drug or solvent was added. Flagella were observed every 20 to 30 minutes by fixing cells with 6.67 × 10^-3^% formaldehyde, a concentration that preserved flagella during the 20-minute observation period. Flagella were observed with the 100× objective and acquired using AxioVision software on an Axiovert 200 M microscope (Carl Zeiss).

### HPLC analysis

A 100 ml culture was grown for four days, spun down and resuspended in 20 ml fresh TAP. Aliquots (1 ml) were treated with drug or solvent for 3 h. Cells were then washed three times with TAP, resuspended in 50 µl TAP media, transferred to clean 1.5 ml tubes and stored frozen at -20°C. The samples were later lysed with 50 µl SDS-EB buffer (2% SDS, 400 mM NaCl, 40 mM EDTA, 100 mM Tris-HCl, pH 8.0) and incubated at 60°C for 1 h. Samples were frozen at -80°C and later processed on HPLC as described [37].

## Abbreviations

DMSO: dimethyl sulfoxide; HPLC: high-pressure liquid chromatography; OD: optical density.

## Competing interests

The authors declare that they have no competing interests.

## Authors' contributions

SEA carried out the study. CL performed screening and deflagellation assays. KL performed screening and motility/phototaxis assays. AM performed photosynthesis retests and HPLC assays. AS and IW performed cheminformatic analysis and web development. MU set up initial experiments and robotics. MP assisted with data collection and developed YG software. SEA and CN conceived of the study and wrote the manuscript. All authors read and approved the manuscript.

## Supplementary Material

Additional file 1 **Supplemental Figures S1, S2, S3, and S4**. Click here for file

Additional file 2 **Supplemental Tables S1 and S2**. Proschold T: **Portrait of a Species: Chlamydomonas reinhardtii**. *Genetics *2005, **170:**1601-1610. Ehler L, Holmes J: **Loss of spatial control of the mitotic spindle apparatus in a Chlamydomonas reinhardtii mutant strain lacking basal bodies**. *Genetics *1995, **141:**945-960. Goodenough UW, StClair HS: **BALD-2: a mutation affecting the formation of doublet and triplet sets of microtubules in Chlamydomonas reinhardtii**. *Journal of Cell Biology *1975, **66:**480-491. Luck D, Piperno G, Ramanis Z, Huang B: **Flagellar mutants of Chlamydomonas: studies of radial spoke-defective strains by dikaryon and revertant analysis**. *Proceedings of the National Academy of Sciences of the United States of America *1977, **74:**3456-3460. Piperno G, Huang B, Luck DJ: **Two-dimensional analysis of flagellar proteins from wild-type and paralyzed mutants of Chlamydomonas reinhardtii**. *Proceedings of the National Academy of Sciences of the United States of America *1977, **74:**1600-1604. Galloway RE, Mets L: **Non-Mendelian inheritance of 3-(3, 4-dichlorophenyl)-1, 1-dimethylurea-resistant thylakoid membrane properties in Chlamydomonas**. *Plant Physiology *1982, **70:**1673. Click here for file
