# Supplementary material for: A phenotypic screening platform to identify small molecule modulators of Chlamydomonas reinhardtii growth, motility and photosynthesis
Source: Genome Biol. 2012 Nov 18;13(11):R105. doi: 10.1186/gb-2012-13-11-r105 (PMC3580497; doi:10.1186/gb-2012-13-11-r105)

Additional file 1

Figure S1A and B. DMSO dose response. (A) *Chlamydomonas* were spotted in 10-fold dilutions onto TAP agar plates with increasing concentrations of DMSO. (B) Growth was assayed in liquid TAP media by inoculating $1.5X10^5$ cells/ml with increasing concentrations of DMSO.

Figure S2. Chemical screening in 1536-well plates. Screen shot of Yeast Grower software output showing growth curves for one quarter of a 1536-well plate with 9 μl of *Chlamydomonas* liquid culture per well. (Inset) Dose response curve of fluperlapine treated cells obtained from a 1536-well plate growth assay.

Figure S3. HPLC analysis of drug uptake. (Top Panel) Drug standard of 7.5 nmol oryzalin displaying a retention time of 4.6 minutes (green box). (Middle and Bottom Panels) Cell lysate of *Chlamydomonas* incubated with solvent or drug for 3 hours. Initial peak (blue box; retention time 0.8 min.) corresponds to cellular debris. (Middle Panel) Solvent treated cells show no peak at the retention time for oryzalin (green box). (Bottom Panel) Oryzalin treated cells show a peak at the same retention time as the oryzalin standard (green box).

Figure S4. Effects of vinpocetine on *Chlamydomonas* phototaxis. (A) DMSO control. Untreated cells display negative phototaxis (movement away from light). (B) Vinpocetine treated. Cells treated with 2.3 μM vinpocetine display a positive phototaxis response. Arrows indicate direction of illumination.

Figure S1

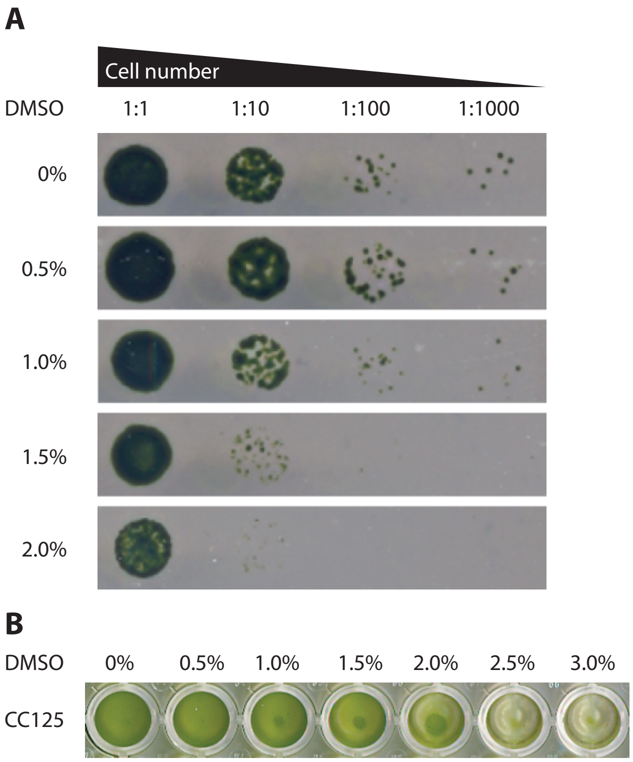
A
Cell number
DMSO
1:1
1:10
1:100
1:1000
0%
0.5%
1.0%
1.5%
2.0%
B
DMSO
0%
0.5%
1.0%
1.5%
2.0%
2.5%
3.0%
CC125

Figure S2

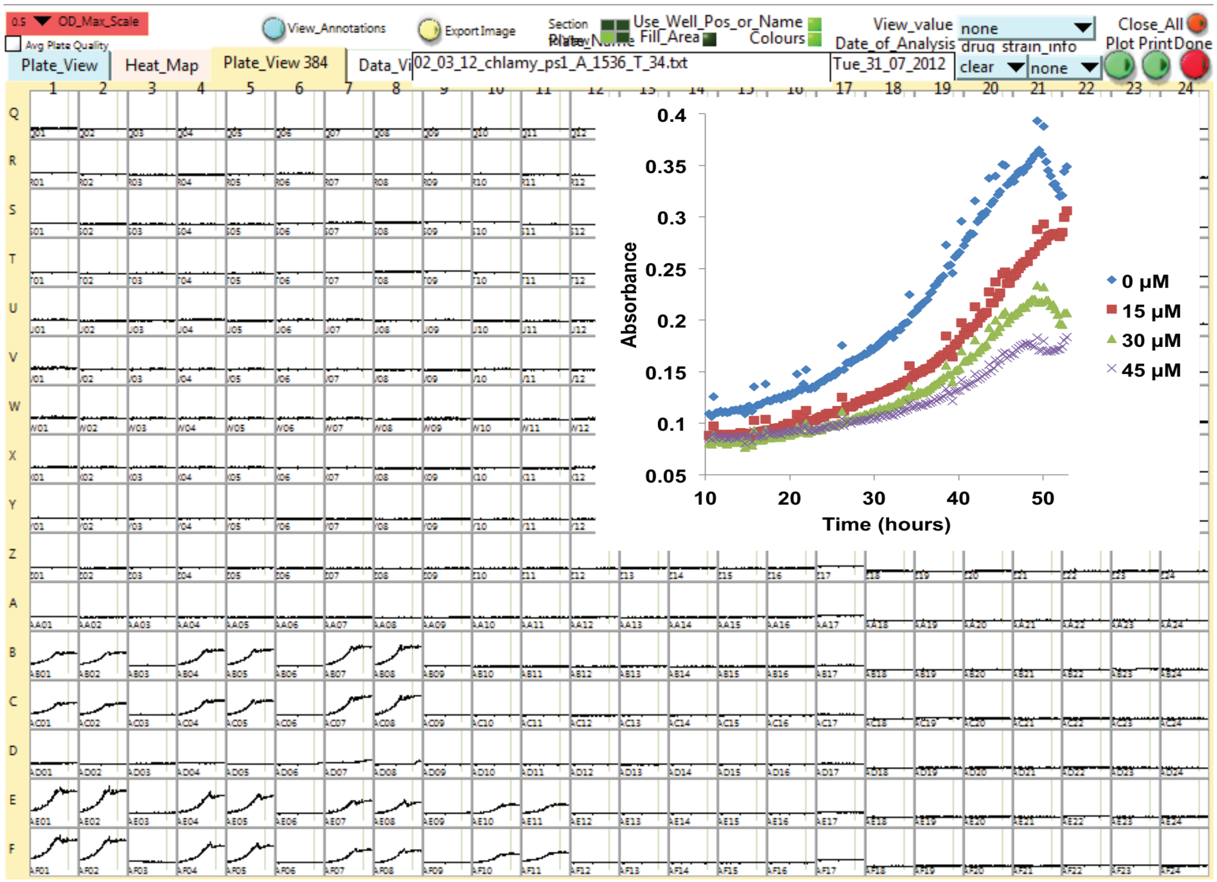
Absorbance
Time (hours)
0 µM
15 µM
30 µM
45 µM
Plate_View
Heat_Map
Plate_View 384
02_03_12_chlamy_ps1_A_1536_T_34.txt
Tue_31_07_2012

Figure S3

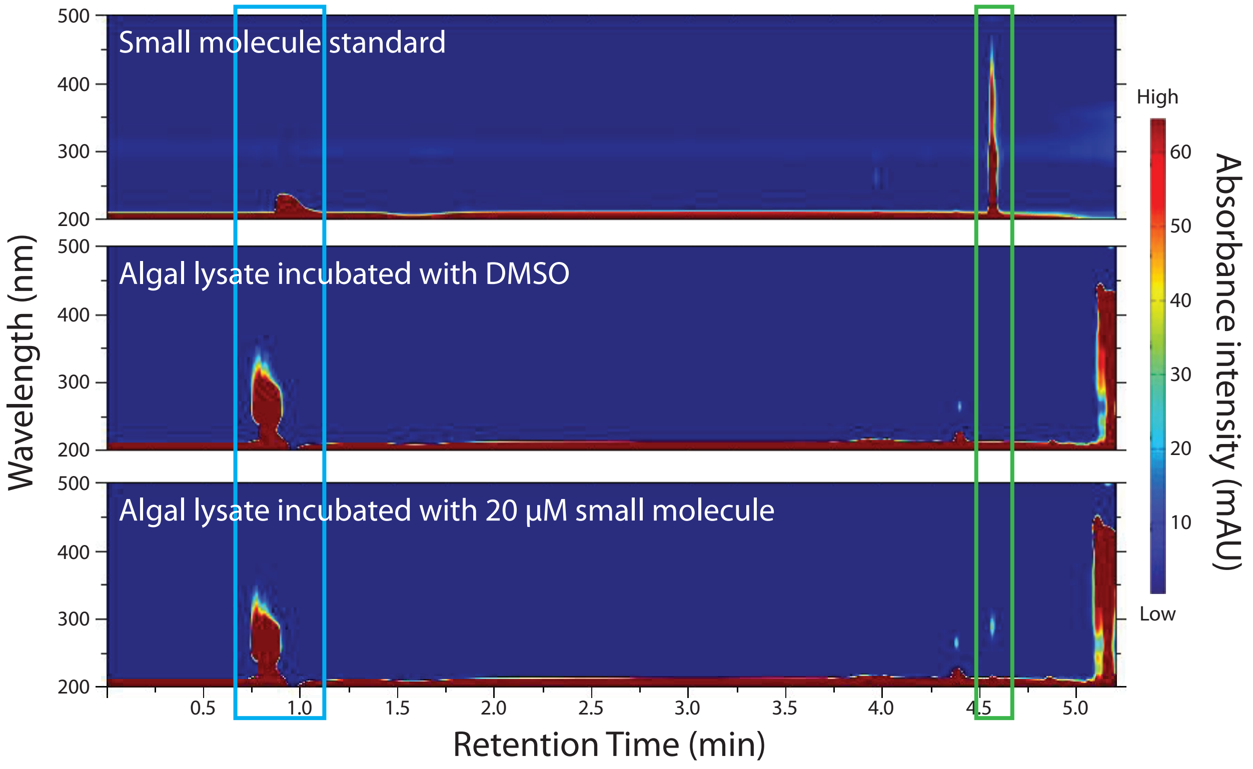

Small molecule standard
Algal lysate incubated with DMSO
Algal lysate incubated with 20 µM small molecule
500
400
300
200
Wavelength (nm)
0.5
1.0
1.5
2.0
2.5
3.0
3.5
4.0
4.5
5.0
Retention Time (min)
High
60
50
40
30
20
10
Low
Absorbance intensity (mAU)

Figure S4

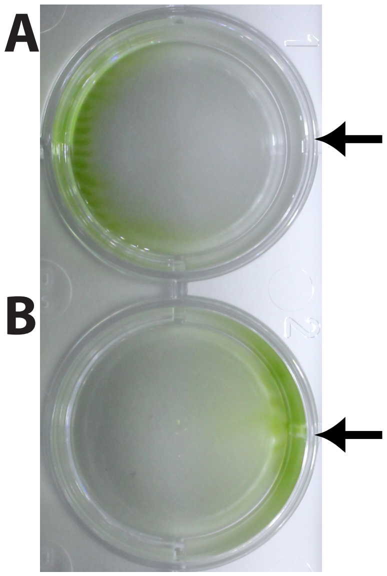

Supplement: Additional file 1 — Supplemental Figures S1, S2, S3, and S4. [file gb-2012-13-11-r105-S1.PDF]
