# Supplementary material for: A phenotypic screening platform to identify small molecule modulators of Chlamydomonas reinhardtii growth, motility and photosynthesis
Source: Genome Biol. 2012 Nov 18;13(11):R105. doi: 10.1186/gb-2012-13-11-r105 (PMC3580497; doi:10.1186/gb-2012-13-11-r105)
Supplement: Additional file 2 — Supplemental Tables S1 and S2. Proschold T: Portrait of a Species: Chlamydomonas reinhardtii. Genetics 2005, 170:1601-1610. Ehler L, Holmes J: Loss of spatial control of the mitotic spindle apparatus in a Chlamydomonas reinhardtii mutant strain lacking basal bodies. Genetics 1995, 141:945-960. Goodenough UW, StClair HS: BALD-2: a mutation affecting the formation of doublet and triplet sets of microtubules in Chlamydomonas reinhardtii. Journal of Cell Biology 1975, 66:480-491. Luck D, Piperno G, Ramanis Z, Huang B: Flagellar mutants of Chlamydomonas: studies of radial spoke-defective strains by dikaryon and revertant analysis. Proceedings of the National Academy of Sciences of the United States of America 1977, 74:3456-3460. Piperno G, Huang B, Luck DJ: Two-dimensional analysis of flagellar proteins from wild-type and paralyzed mutants of Chlamydomonas reinhardtii. Proceedings of the National Academy of Sciences of the United States of America 1977, 74:1600-1604. Galloway RE, Mets L: Non-Mendelian inheritance of 3-(3, 4-dichlorophenyl)-1, 1-dimethylurea-resistant thylakoid membrane properties in Chlamydomonas. Plant Physiology 1982, 70:1673. [file gb-2012-13-11-r105-S2.DOCX]

**Additional file 2**

**Table S1. Strains used in this study.**

| Stock Center ID | Genotype | Reference |
| --- | --- | --- |
| CC-125 mt+ | *wild-type nit1, nit2* | *[*[*7*](#_ENREF_72)*3]* |
| CC-478 mt+ | *bld2 (tue)* | *[*[*74*](#_ENREF_74)*, 75]* |
| CC1032 mt+ | *pf14 (rsp3)* | *[*[*76*](#_ENREF_76)*, 77]* |
| CC1403 mt+ | *dr-u-2 (psbA V219I)* | *[*[*44*](#_ENREF_44)*,* [*78*](#_ENREF_77)*]* |

**Table S2. Chemical libraries screened in this study.**

| Chemical Library | Supplier | Screened | Notes |
| --- | --- | --- | --- |
| FDA BML-640 | Enzo Life Sciences, Farmingdale, NY | 640 |  |
| TimTec NPL-280 | TimTec LLC, Newark, DE | 280 |  |
| Yactives | Yactives, ChemBridge and ChemDiv, San Diego, CA. Total size 7476. | 4357 | Library derived from ChemBridge (NOVACore and DIVERSet, San Diego, CA, USA) and ChemDiv (Divers, San Diego, CA, USA) |
| NOVACore | NOVACore, ChemBridge, San Diego, CA | 168 |  |
